# Natural history of cardiac involvement in women carrying pathogenic *DMD* gene variants: a 7-year longitudinal study

**DOI:** 10.1007/s00415-026-13856-4

**Published:** 2026-05-20

**Authors:** Zhe Lyu, Heini Joensen, Nanna Scharff Poulsen, Maria Buhl Hjortrup, Anna Slipsager, Bjørk Teitsdóttir, Freja Fornander, Tuva Åsatun Solheim, Adam Espe Hansen, Niels Grove Vejlstrup, John Vissing

**Affiliations:** 1 Copenhagen Neuromuscular Center, Rigshospitalet, University of Copenhagen, Copenhagen, Denmark; 2 Department of Cardiology, The Heart Center, Rigshospitalet, Copenhagen, Denmark; 3 Department of Radiology, Rigshospitalet, Copenhagen, Denmark

**Keywords:** Arrhythmia, CMR, *DMD* gene variants, Ejection fraction, Myocardial fibrosis

## Abstract

**Background:**

Cardiac involvement is increasingly recognized in women carrying pathogenic variants in the dystrophin (*DMD)* gene, both Duchenne and Becker-associated, but the long-term natural history of cardiac structure, function, and conduction abnormalities in this group is not well understood. We conducted a prospective 7-year follow-up study to investigate the evolution of ventricular function, myocardial fibrosis, and arrhythmias.

**Methods:**

34 women with confirmed pathogenic *DMD* gene variants (19 predicted to cause Duchenne muscular dystrophy and 15 Becker muscular dystrophy) underwent assessments after a mean of 7 years. Evaluations included cardiac magnetic resonance imaging with late gadolinium enhancement, 24-h Holter monitoring, 12-lead electrocardiography (ECG), cardiac biomarkers, and clinical examinations.

**Results:**

At the group level, left ventricular ejection fraction (LVEF) remained stable over follow-up (*p* = 0.403). Six women had a ≥ 5% change in LVEF: three improved with treatment, and three declined by 5–8%. Fibrosis was present in 9 women at both baseline and follow-up; 3 showed visual progression, and no new cases were identified. Although the median ventricular premature contractions per hour (VPC/h) increased from 0.23/h to 6.38/h (*p* < 0.001), this increase is likely not clinically meaningful as age-related increases in ventricular ectopy are also seen in healthy populations, and high-burden arrhythmias—according to prespecified thresholds—remained uncommon.

ECG parameters remained stable (PR, QRS, and QTc unchanged), with no new bundle-branch blocks or high-grade atrioventricular block detected. No association was observed between LVEF, VPC/h, and supraventricular premature contraction per hour over the 7-year follow-up period.

**Conclusions:**

Over 7 years, women carrying pathogenic *DMD* gene variants have very little or no progression of cardiac findings (LVEF, fibrosis, and arrhythmias). Given these findings, frequent routine rhythm monitoring is not indicated. Follow-up should be risk-stratified: closer for women with symptoms, baseline high-burden ectopy, reduced LVEF, or pre-existing fibrosis; others can be reviewed at extended intervals.

## Introduction

Duchenne muscular dystrophy (DMD) and Becker muscular dystrophy (BMD) are X-linked recessive disorders caused by pathogenic variants in the dystrophin (*DMD*) gene, leading to progressive muscle degeneration, cardiomyopathy, and respiratory failure in affected men [1].

While women carrying pathogenic *DMD* gene variants are traditionally considered asymptomatic, some exhibit skeletal muscle and cardiac involvement [2–5].

Cardiac abnormalities are common in these women, with reported prevalences ranging widely across studies (3%–84.3%) because of heterogeneous age distribution and assessment methods [6, 7].

Manifestations include myocardial fibrosis, left ventricular dysfunction, dilated cardiomyopathy, and conduction abnormalities with arrhythmias, even in women with no or only mild muscle weakness [8, 9].

In a cardiac magnetic resonance imaging (CMR)-based cohort, around 47% of women had reduced left ventricular ejection fraction (LVEF) and/or late gadolinium enhancement (LGE), even in the absence of overt muscle weakness [8]. Another prospective cohort study reported that 49% of women with *DMD* gene variants had myocardial fibrosis on CMR versus 0% in age-matched healthy controls, despite normal skeletal muscle function [9]. Together, these findings support the need for cardiac screening even when neuromuscular symptoms are absent.

Our previous cross-sectional study of women carrying pathogenic *DMD* gene variants showed that more than 2/3 of the women had structural and/or functional cardiac abnormalities, raising concern for subclinical cardiac involvement and motivating longitudinal follow-up to define trajectories and inform surveillance strategies.

However, the natural history of cardiac involvement in this group remains largely unknown.

Although early imaging abnormalities, such as LGE, increased left ventricular end-systolic volume, and reduced global longitudinal strain, are observed in some women, it remains uncertain whether these subclinical findings are consistent over time or can reliably predict future dysfunction. This uncertainty likely contributes to the absence of standardized guidelines to date for long-term cardiac monitoring in this group.

To address this knowledge gap, we conducted a prospective 7-year follow-up study in women carrying pathogenic *DMD* gene variants. Using serial assessments including CMR, 24-h Holter monitoring, and electrocardiography (ECG), we characterized trajectories of myocardial fibrosis, cardiac structure and function, and conduction abnormalities and arrhythmias over time and explored associations with skeletal muscle involvement.

## Methods

### Ethics approval

The study was approved by the Danish National Committee on Health Research Ethics (Approval number: H-23007727) and registered at ClinicalTrials.gov (Identifier: NCT05715957). All participants provided written informed consent. The study was conducted in accordance with the Declaration of Helsinki.

### Study design and participants

The study was a longitudinal follow-up study.

We invited all 53 women from the baseline cohort [10] with a confirmed pathogenic variant in the *DMD* gene to a 7-year follow-up evaluation. Assessments included CMR (with LGE when possible), 24-h Holter monitoring, 12-lead ECG, blood sampling, muscle magnetic resonance imaging (MRI), muscle strength testing, and a review of medical history.

Participants were excluded from CMR if they had MRI contraindications (e.g., pacemaker, MR-incompatible metallic implants, or severe claustrophobia).

Contraindications to gadolinium (e.g., estimated glomerular filtration rate (eGFR) < 30 mL/min/1.73 m [2], prior severe contrast reaction, pregnancy, or breastfeeding) precluded contrast; in such cases, a noncontrast CMR protocol was used.

Participants who did not undergo CMR still completed all other study assessments.

### Cardiac magnetic resonance imaging

Follow-up CMR was performed on a 3.0 Tesla scanner (MAGNETOM Vida, Siemens, Erlangen, Germany), whereas baseline CMR had been acquired on an older 3.0 Tesla scanner (MAGNETOM Verio, Siemens, Erlangen, Germany).

At the same field strength (3.0 Tesla), acquisition parameters, slice planes, sequences, and analysis procedures were harmonized with the baseline study [10] to ensure methodological consistency and enable direct comparability across scanners and time points.

Participants were scanned supine, head-first, using a cardiac coil.

Sequences included cine steady-state free precession imaging, modified Look-Locker inversion recovery (MOLLI) (pre- and post-contrast), and LGE.

Left and right ventricular volumes, -function, and -mass, as well as left atrial volume, were measured using the same anatomical landmarks and timing phases as in the baseline study. T1 maps were generated from MOLLI sequences acquired before and after contrast administration to construct relaxation curves and parametric maps.

Contrast-enhanced imaging was performed 7 min after intravenous gadobutrol (Gadovist, Bayer Schering, Germany). Gadolinium dosage was adjusted according to renal function: 0.1 mmol/kg for eGFR 30–60 mL/min/1.73 m^2^ and 0.15 mmol/kg for eGFR ≥ 60 mL/min/1.73 m [2]. In all cases, the maximum dose did not exceed 15 mL, regardless of body weight.

LGE was evaluated visually and quantified using a signal intensity threshold of > 6 standard deviations above that of remote myocardium*,* defined as a region without visual evidence of fibrosis or abnormal enhancement. LGE localization was assessed visually by left ventricular wall distribution, and extent was summarized semiquantitatively as the number of involved wall segments. Visual progression was additionally noted when the area of LGE appeared more extensive at follow-up, even without an increase in the number of involved wall segments.

Hematocrit was measured on the day of CMR, and myocardial extracellular volume (ECV) was calculated from native and post-contrast myocardial and blood-pool T1 values, together with hematocrit, using the published Eq.  [11].

CMR abnormalities were defined according to current CMR guidelines [12–17]:

- Ventricular function:
  LVEF < 56%;
  Right ventricular ejection fraction (RVEF) < 49%
- Chamber enlargement:
  Left ventricular end-diastolic volume (LVEDV) > 143 mL;
  Indexed LVEDV >108 mL/m^2^;
  Indexed left ventricular mass > 74 g/m^2^;
  Indexed left atrial (LA) maximum volume > 53 mL/m^2^;
  Indexed right ventricular end-diastolic volume (RVEDV) > 153 mL/m^2^
- Myocardial tissue:
  LGE presence;
  ECV > 30%;
  Native T1 relaxation time > 1269 ms

- Left ventricular hypertrophy: Wall thickness ≥ 12 mm

Changes in LVEF were considered clinically meaningful when an increase of ≥ 5% was found, whereas a decrease of ≥ 5% with symptoms, or ≥ 10% without symptoms, that resulted in a final LVEF below the normal range was considered clinically meaningful dysfunction [18, 19].

Quantitative image analysis was performed using cvi42 (version 6.2.2; Circle Cardiovascular Imaging, Calgary, Canada). All CMR images were analyzed by ZL, followed by review and confirmation by either NGV (for fibrosis) or MBH (for LVEF). Reviewers were blinded to participants’ clinical information and scan timepoints (baseline vs. follow-up).

### Holter monitoring

24-h Holter monitoring was obtained at follow-up from two sources: 1) hospital summary reports, or 2) study recordings using the Bittium Faros Manager device (Bittium Biosignals Ltd, Kuopio, Finland; Version 3.4.1).

Study recordings were analyzed in Bittium Cardiac Navigator™ software (Version 1.5.6) by ZL and NSP, both of whom were blinded to participants’ clinical status and baseline data, and the same diagnostic thresholds as at baseline were applied.

Holter monitoring was considered abnormal if any of the following were present [20]:

- Atrioventricular block (AVB) degrees I–III
- Atrial arrhythmias: atrial fibrillation or atrial flutter
- Supraventricular tachyarrhythmia (SVT): > 30 supraventricular premature contractions per hour (SVPC/h) or run of ≥ 20 SVPC
- Frequent ventricular premature contraction (VPC) > 30/h
- Nonsustained ventricular tachycardia (NSVT) ≥ 3 beats at ≥ 100 bpm
- Presence of supraventricular or ventricular couplets and/or triplets

### Electrocardiography

A standard 12-lead ECG was performed at follow-up using the same equipment (GE Healthcare MAC 3500, Freiburg, Germany) as in the baseline study [10].

All ECG recordings, both at baseline and follow-up, were evaluated by ZL using the same diagnostic criteria established in the initial assessment, ensuring consistent and reliable comparison over time.

ECG findings were classified as abnormal in the presence of any of the following [21, 22]:

- Atrial arrhythmias: atrial fibrillation and atrial flutter
- AVB: first-degree (PR interval > 200 ms), second-degree (Mobitz I (Wenckebach) and Mobitz II), or third-degree (complete AV dissociation)
- Intraventricular conduction abnormalities: QRS duration (> 120 ms), corrected QT interval (QTc > 470 ms), or incomplete right bundle branch block (IRBBB)
- DMD-associated electrocardiographic markers: R-wave amplitude > 4 mm in leads V1–V2, elevated R/S ratio in V1 or V2 in the absence of right bundle branch block (RBBB), pathological Q waves (> 0.2 mV) in lateral (I, aVL, V5, V6) or inferior (II, III, aVF) leads, complete or incomplete left bundle branch block (LBBB or ILBBB), or complete RBBB

### Cardiac biomarker

Blood samples were analyzed for creatine kinase (CK), CK-MB, pro-brain natriuretic peptide (pro-BNP), and troponin T.

CK-MB was considered abnormal when the level was above the reference range (> 4.0 µg/L), and the CK-MB/total CK ratio was ≥ 3%, which was interpreted as indicative of cardiac rather than skeletal muscle involvement [23].

ProBNP was considered abnormal when it exceeded the age-specific upper reference limit: > 15.3 pmol/L (18–44 years), > 29.4 pmol/L (45–54 years), > 33.9 pmol/L (55–64 years), > 35.5 pmol/L (65–74 years), and > 87.1 pmol/L (≥ 75 years).

Troponin T was considered abnormal at concentrations ≥ 14 ng/L.

### Muscle magnetic resonance imaging and muscle strength

Whole-body muscle MRI was performed on the same 3.0 Tesla scanners as CMR using harmonized T1-weighted and Dixon sequences, as previously described [24].

Participants were scanned in supine, head-first position with a peripheral angiographic coil and a body matrix coil.

Muscle fat fraction (FF) was manually analyzed at the mid-thigh level using Horos™ software (version 4.0). Cross-sectional slices were acquired at 50% of the femur length, as in another MRI study [25].

All images were analyzed by ZL and reviewed by NSP, both investigators were blinded to the participants' clinical information and scan time points.

The muscle FF was calculated as: *FF* = *(I *_*fat*_* / I *_*in*_*)* × *100,* where *I *_*fat*_ is the fat signal intensity and *I *_*in*_ is the in-phase signal intensity.

Manual muscle strength was assessed bilaterally using the Medical Research Council (MRC) scale, testing elbow flexion and extension, wrist palmar flexion and dorsiflexion, finger flexion and extension, knee flexion and extension, and ankle dorsiflexion and plantarflexion.

The MRC scale ranges from 0 (no contraction) to 5 (normal strength). Assessments were performed by trained clinicians using standardized positioning to ensure consistency across participants.2.8) Statistical analyses.

Analyses were conducted using GraphPad Prism version 9 (GraphPad Software, San Diego, CA, USA).

Normality of continuous variables was assessed using the Shapiro–Wilk test and by visual inspection of histograms and Q–Q plots. For normally distributed data, paired *t* tests were used to compare baseline and follow-up measurements. For non-normally distributed data, Wilcoxon matched-pairs signed-rank tests were applied.

Descriptive data is presented as mean ± standard deviation (SD) for normally distributed variables and as median with interquartile range (IQR) for non-normally distributed variables. The results are reported as mean or median difference along with the (bootstrapped) 95% confidence interval (CI), where applicable.

Linear regression analysis was used to evaluate the correlations between LVEF and muscle FF at follow-up, between change in LVEF (ΔLVEF = follow-up LVEF − baseline LVEF) and changes in muscle FF (ΔFF = follow-up FF − baseline FF), and between changes in VPC/h and SVPC/h and muscle ΔFF.

Statistical significance was defined as *p* < 0.05.

## Results

### Characteristics of women with pathogenic *DMD* gene variants

Of the 53 women re-invited, 34 (64%) completed the follow-up visit. The mean follow-up time was 7.1 years (range: 6.5–7.7 years).

**Fig. 1:**
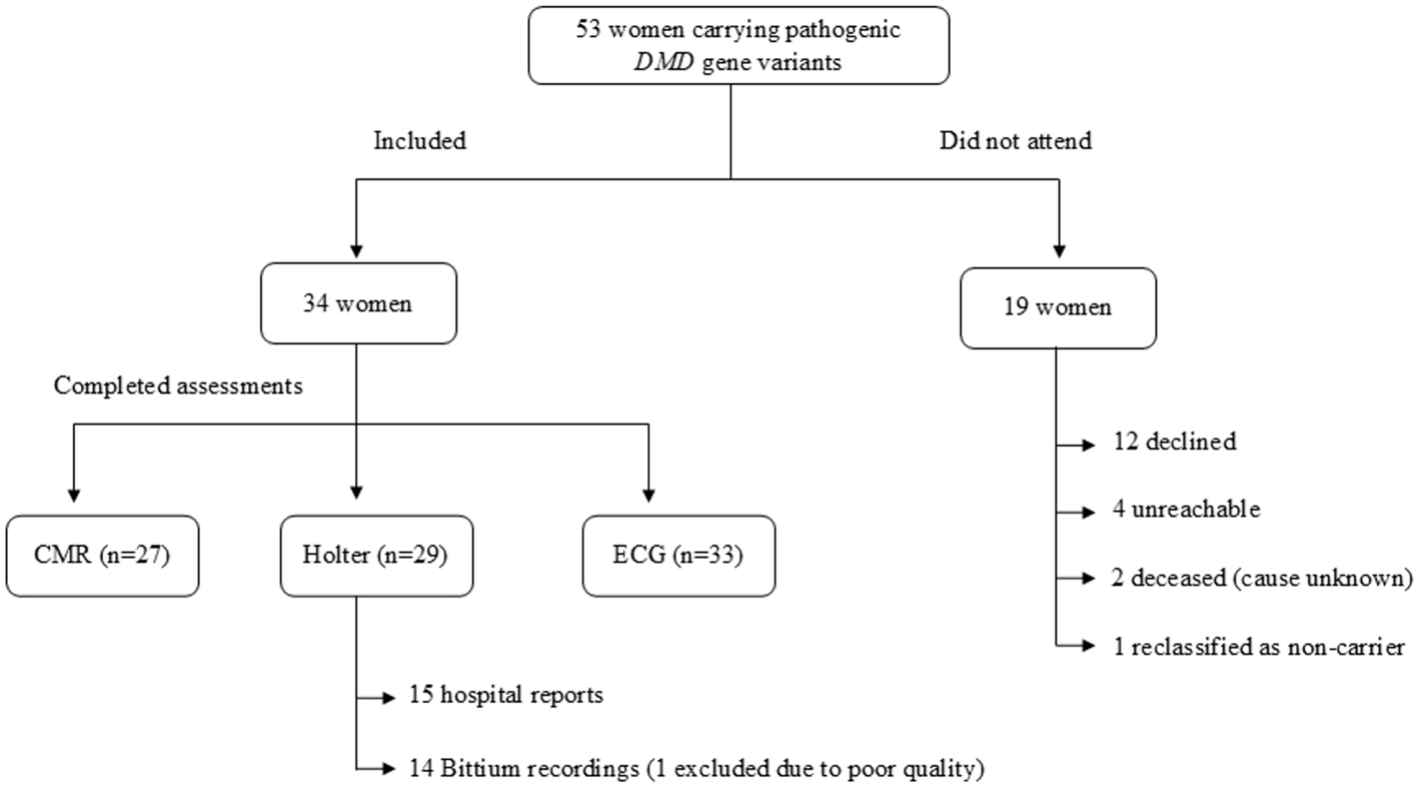
Participant flow and completion of cardiac examinations at the 7-year follow-up. CMR was performed in 27 participants; 25 received gadolinium contrast, and 2 declined. Seven participants did not undergo CMR: 2 for clinical reasons (pregnancy, n = 1; recent myocardial infarction, n = 1) and 5 declined. *CMR* cardiac magnetic resonance imaging, *DMD* gene, dystrophin gene; *ECG* electrocardiogram.

Among these 34 participants, six had reduced LVEF, and seven had arrhythmias. Completion of CMR, Holter, and ECG at follow-up is shown in Fig. 1.

Nineteen (36%) did not attend follow-up. At baseline, 2/19 had reduced LVEF, and 5/19 had arrhythmias (two with SVPC ≥ 30/h, one with SVPC run ≥ 20, two with VPC ≥ 30/h, and one with NSVT).

The mean age at follow-up was 54.6 years (SD 13.6); 19 variants predicted DMD and 15 BMD. Nine participants (26%) initiated heart failure or antihypertensive treatment between baseline and follow-up, including angiotensin-converting enzyme inhibitors, angiotensin receptor blockers, and beta-blockers **(**Table 1**).** None of the participants received cardiac medication before baseline.

**Table 1:** Characteristics of the women with pathogenic *DMD* gene variants

Participant ID	Age (y)	Pathogenic variant	Genotype predicting BMD or DMD	ΔLVEF (%)	Cardiac medication initiated between visits
01	68	Del ex. 3–16	DMD	NA	None
02	50	Del ex. 3–9	BMD	-8.2	None
03	46	Del ex. 45–50	DMD	-6.3	None
04	32	c.3806delA	DMD	-2.2	None
05	67	c.2622 + 1G- > A	DMD	NA	None
06	37	Del ex. 48	BMD	-2.9	None
07	77	Del ex. 46–55	DMD	3.3	Losartan
08	67	Del ex. 45–52	DMD	NA	None
09	73	Del ex. 51	BMD	-0.3	None
10	71	Del ex. 37–43	BMD	-0.1	None
11	57	Del ex. 14–21	DMD	-2.0	None
12	44	Del ex. 51	BMD	4.2	None
13	52	c.5632C > T p. (Gln1878*)	BMD	15.1	Metoprolol; Ramipril
14	48	Del ex. 46–50	DMD	-0.3	None
15	52	Del ex. 45–48	BMD	3.0	None
16	48	Del ex. 45–47	BMD	2.6	None
17	59	Del ex. 47–51	DMD	3.3	None
18	40	Del ex. 45–48	BMD	4.8	None
19	70	Del ex. 45–49	BMD	NA	Sacubitril; Eplerenone; Metoprolol; Dapagliflozin
20	62	Dup ex. 8–18 and 41–45	DMD	-1.2	None
21	53	Del ex. 13	BMD	7.7	Losartan
22	36	c.1114.delG	DMD	-5.4	Ramipril; Carvedilol
23	51	Del ex. 45–47	BMD	-3.0	None
24	40	Del ex. 13	BMD	NA	None
25	39	c.10108C > T p. (Arg3370*)	DMD	-1.9	None
26	64	Dup ex. 18–19	DMD	3.8	Losartan
27	44	Del ex. 45	DMD	-0.7	None
28	73	Del ex. 18–44. c.8038C > T	BMD	NA	Metoprolol
29	47	c.8038C > T p. (Arg2680*)	DMD	-2.7	None
30	50	c.3295C > T p. (Gln1099*)	DMD	7.6	Amlodipine
31	40	Del ex. 51	DMD	-3.6	Losartan
32	64	Del ex. 44–48	BMD	4.4	None
33	87	Del ex. 53	DMD	NA	None
34	48	Del ex. 44	DMD	2.9	None

* Denotes a nonsense mutation that introduces a premature stop codon, leading to a truncated protein product. *BMD* Becker muscular dystrophy, *DMD* Duchenne muscular dystrophy, *LVEF* left ventricular ejection fraction, *ΔLVEF* follow-up LVEF − baseline LVEF), *NA* not available, *y* year

During follow-up, three participants (9%) who had exertional dyspnea at baseline, reported worsened symptoms, and one experienced a myocardial infarction.

### Cardiovascular magnetic resonance imaging

Over the 7-year follow-up period, women carrying pathogenic *DMD* gene variants maintained stable LVEF with no significant changes (mean difference: + 0.81%, 95% CI: − 1.14% to + 2.76%; *p* = 0.403, n = 27). After excluding participants who initiated heart failure or antihypertensive medication, there was no change in LVEF during the 7-year follow-up (mean difference: − 0.33%, 95% CI: − 1.99% to + 1.33%; *p* = 0.683, n = 20).

At baseline, six women had an abnormal LVEF (< 56%). Of these, three still had an abnormal LVEF at follow-up. Only one woman (Participant 22, a DMD carrier) showed a clinically meaningful decline in LVEF > 5%, despite treatment initiated at baseline. In the other two women, LVEF decreased by < 5%.

The remaining three showed a clinically meaningful improvement (≥ 5%), with LVEF returning to the normal range after initiation of cardiac therapy.

Two additional women (Participants 02 and 03) showed a below-clinically meaningful decrease in LVEF of ≥ 5%, but within the normal range (going from 66% to a little below 60%).

The remaining 19 women (78%) maintained stable LVEF values (< 5% change) between visits.

Myocardial fibrosis, detected by LGE, was present in nine women at both baseline and follow-up. No new cases were identified during the study period. Notably, seven of the nine women had normal LVEF, whereas two had reduced function.

LGE was located in the inferolateral or lateral–inferolateral left ventricular wall in all affected women. Segment-based localization and the number of involved wall segments remained stable over time, with all cases limited to one involved segment.

**Fig. 2:**
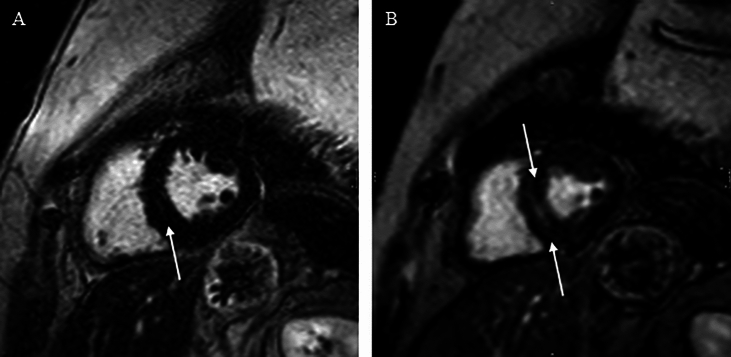
Cardiac magnetic resonance imaging with late gadolinium enhancement in Participant 09 at baseline and 7-year follow-up. Short-axis images are shown (**A**: baseline, **B**: follow-up). Mild subepicardial enhancement is seen in the lateral wall at baseline, with progression at follow-up (white arrows), consistent with advancing myocardial fibrosis.

However, in three of the nine women, LGE appeared visually more extensive at follow-up, with no increase in the number of involved wall segments; image quality was too low to allow formal quantification (Fig. 2). Other CMR-assessed abnormalities in cardiac structure, including left ventricular hypertrophy, changes in ventricular volumes and function, remained stable from baseline to follow-up, with no major progression observed (Tables 2 and 3).

**Table 2:** CMR abnormalities in women carrying pathogenic *DMD* gene variants at baseline and follow-up

	Baseline	Follow-up
Left ventricle (n = 27)		
LVEF (**> **55%)	21 (78%)	24 (89%)
LVEF decreased mildly (45–55%)	5 (19%)	2 (7%)
LVEF decreased moderately (35–44%)	1 (4%)	1 (4%)
LVEDV increased mildly (144–179 ml)	10 (37%)	11 (41%)
LVEDV increased moderately (180–220 ml)	3 (11%)	1 (4%)
LVEDV indexed increased mildly (109 –130 ml/m^2^)	1 (4%)	1 (4%)
LV mass indexed increased (> 74 g/m^2^)	0 (0%)	0 (0%)
Left ventricular hypertrophy	4 (15%)	4 (15%)
Right ventricle (n = 27)		
RVEF decreased mildly (37–48%)	3 (11%)	3 (11%)
RV volume indexed increased mildly (> 153 ml/m^2^)	3 (11%)	1 (4%)
Left atrium (n = 27)		
LA max volume indexed increased (> 53 ml/m^2^)	0 (0%)	0 (0%)
Fibrosis		
Any LGE (n = 25)	9 (36%)	9(36%)
Native T1 > 1269 ms (n = 25)	1 (4%)	1 (4%)
ECV increased in MOLLI images (> 30%) (n = 22)	2 (9%)	2 (9%)

Normal reference values: LVEF > 55%, RVEF ≥ 49%, LVEDV ≤ 143 mL, LVEDVi ≤ 108 mL/m^2^, indexed LV mass ≤ 74 g/m^2^, indexed LA maximum volume ≤ 53 mL/m^2^, RVEDVi ≤ 153 mL/m^2^, ECV ≤ 30%, native T1 < 1269 ms

*ECV* extracellular volume, *LA* left atrium, *LV* left ventricle, *LVEDV* left ventricular end-diastolic volume, *LGE* late gadolinium enhancement, *LVEF* left ventricular ejection fraction, *MOLLI* modified look-locker inversion recovery, n number, *RV* right ventricle, *RVEF* right ventricular ejection fraction

**Table 3:** Changes in CMR imaging findings at baseline and follow-up in women with pathogenic *DMD* gene variants

Participant ID	LVEF	LVEDV	LVEDV indexed	Fibrosis	ECV (> 30%)	Progression
02	↓	↔	↔	N → N	N → N	N
03	↓	↔	↔	N → N	N → N	N
04	↔	↑	↔	Y → Y	Y → Y	N
06	↔	↔	↔	N → N	N → N	N
07*	↔	↔	↔	N → N	N → N	N
09	↔	↔	↔	Y → Y	NA	Y
10	↔	↔	↔	N → N	N → N	N
11	↔	↓	↔	Y → Y	N → N	N
12	↔	↓	↔	N → N	N → N	N
13*	↑	↔	↔	Y → Y	Y → Y	N
14	↔	↔	↔	N → N	N → N	N
15	↔	↔	↔	N → N	N → N	N
16	↔	↔	↔	N → N	N → N	N
17	↔	↔	↔	Y → Y	NA	N
18	↔	↔	↔	N → N	NA	N
20	↔	↔	↔	Y → Y	N → N	Y
21*	↑	↓	↔	N → N	N → N	N
22*	↓	↓	↔	Y → Y	N → N	Y
23	↔	↓	↔	N → N	N → N	N
25	↔	↔	↔	Y → Y	N → N	Y
26*	↔	↔	↔	NA	NA	N
27	↔	↑	↔	Y → Y	N → N	N
29	↔	↔	↔	N → N	N → N	N
30*	↑	↔	↔	N → N	N → N	N
31*	↔	↔	↔	NA	NA	N
32	↔	↔	↔	N → N	N → N	N
34	↔	↔	↔	N → N	N → N	N

*Indicates participants who initiated medications between visits that may affect LVEF

↑/↓: change ≥ 5%; ↔ : no change or change < 5%.

For fibrosis and ECV: N → N = normal, Y → Y = persistent.

Progression was defined as: an LVEF decrease of ≥ 5% with symptoms, or ≥ 10% without symptoms; new or worsened fibrosis; or new ECV > 30%.

*ECV* extracellular volume fraction, *LVEDV* left ventricular end-diastolic volume, *LVEF* left ventricular ejection fraction, *NA*: Data not available due to artefacts or lack of contrast agent, *N* no, *Y* yes

Overall, LVEDV remained largely stable over follow-up. Although absolute LVEDV was increased in some women, indexed LVEDV was abnormal in only one participant and changed only slightly over time (from 110 mL/m^2^ to 121 mL/m^2^). Therefore, we consider this change to be of limited clinical relevance.

### Holter monitoring

Over the 7-year follow-up period, the proportion of women with Holter abnormalities remained relatively stable, increasing slightly from 34% (10/29) to 45% (13/29).

Overall, increases were mostly within clinical reference ranges, with a few women crossing abnormal thresholds.

High-burden arrhythmias remained uncommon: VPC ≥ 30/h was observed in one participant (1/29) at baseline and in two different participants (2/29) at follow-up. NSVT was present in one participant (1/29) at each time point, but in different individuals.

SVPC > 30/h was observed in one participant at baseline and in a different participant at follow-up (1/29). The baseline case did not receive treatment.

An SVPC run ≥ 20 beats and SVPC > 30/h appeared de novo at follow-up in one participant (Table 4).

**Table 4:** Changes in Holter monitoring findings at baseline and follow-up in women with pathogenic *DMD* gene variants

Holter findings	Baseline (n = 29)	Follow-up (n = 29)
AVB grade I	0 (0%)	1 (3%)
AVB grade II	2 (7%)	1 (3%)
Supraventricular ectopy		
SVPC/h	0.37 (IQR 0.13–1.06)	1.77 (IQR 0.63–4.98)
SVPC > 30/h	1 (3%)	1 (3%)
SVPC runs ≥ 20	0 (0%)	1 (3%)
SVT*	1 (3%)	2 (7%)
Couplets	5 (17%)	8 (28%)
Triplets	4 (14%)	4 (14%)
Ventricular ectopy		
VPC/h	0.23 (IQR 0.04–1.25)	6.38 (IQR 0.85–15.10)
VPC ≥ 30/h	1 (3%)	2 (7%)
Ventricular couplet	2 (7%)	7 (24%)
NSVT	1 (3%)	1 (3%)
Any Holter abnormality	10 (34%)	13 (45%)

*Means SVPC > 30/h or SVPC runs ≥ 20. All continuous values are presented as median (range); all categorical values are presented as numbers (%).*AVB* atrioventricular block, *h* hour, *NSVT* nonsustained ventricular tachycardia, *SVPC/h* supraventricular premature contractions per hour, *SVT* supraventricular tachycardia, *VPC/h* ventricular premature contractions per hour

Below these thresholds, ectopy increased:

Median VPC/h increased from 0.23 /h (IQR 0.04–1.25) at baseline to 6.38 (IQR 0.85–15.10) at follow-up (*p* = 0.000686).

Median SVPC/h increased, nonsignificantly, from 0.37/h (IQR 0.13–1.06) to 1.77/h (IQR 0.63–4.98) (p = 0.078).

Ventricular couplets increased from 7% (2/29) at baseline to 24% (7/29).

Atrioventricular block (AVB) was infrequent: AVB grade I was observed in one woman (3%) at follow-up but was absent at baseline, and this participant (Participant 22) also showed a decline in LVEF on CMR from 44.7% to 39.3%.

AVB grade II was present in two women (7%) at baseline and in one woman (3%) at follow-up.

###  Electrocardiogram

Over the 7-year follow-up period, ECG measurements remained stable, with no clinically meaningful changes observed at either group or individual level.

The QT interval increased significantly from 394.8 ms to 411.4 ms (*p* < 0.001); however, the corrected QT interval (QTc) remained stable (*p* = 0.891), suggesting no relevant change in ventricular repolarization. This QT prolongation may be attributed to a significant reduction in heart rate, which decreased from a mean of 69.5 bpm at baseline to 63.8 bpm at follow-up (*p* = 0.022).

The PR interval (*p* = 0.125) and QRS duration (*p* = 0.324) showed no significant changes between timepoints.

DMD-specific ECG abnormalities were common but did not show clear progression.

At least one abnormality was observed in 61% (20/33) of participants at baseline and in 55% (18/33) at follow-up. The most frequent finding was an R wave > 4 mm in lead V2.

Other abnormalities, including abnormal R/S ratios and isolated Q waves, showed only minor fluctuations over time. Importantly, no new conduction abnormalities, such as right or left bundle branch block, developed during follow-up.

Other ECG findings also remained relatively stable. The proportion of participants with any ECG abnormality was 73% (24/33) at baseline and 67% (22/33) at follow-up **(**Table 5**).**

**Table 5:** Comparison of ECG findings in women carrying pathogenic *DMD* gene variants at baseline and follow-up

	Baseline (n = 33)	Follow-up (n = 33)
**General ECG measurements**	**Mean (SD)**	**Mean (SD)**
Heart rate	69.5 (11.1)	63.8 (9.7)
PR interval (ms)	144.4 (19.3)	147.8 (21.6)
QRS interval (ms)	91.3 (10.6)	89.3 (10.6)
QT interval (ms)	394.8 (24.3)	411.4 (27.6)
QTc interval (ms)	421.4 (20.9)	420.8 (19.8)
**DMD-specific abnormalities**	**Number (%)**	**Number (%)**
R > 4 mm V1	3 (9%)	2 (6%)
R > 4 mm V2	20 (61%)	17 (52%)
R/S > 1 in V1	1 (3%)	2 (6%)
R/S > 1 in V2	3 (9%)	5 (15%)
Q in lateral leads > 0.2 mV	1 (3%)	1 (3%)
Q in inferior leads > 0.2 mV	1 (3%)	1 (3%)
RBBB	1 (3%)	1 (3%)
LBBB	0 (0%)	0 (0%)
Any DMD-specific change	20 (61%)	18 (55%)
**Other measurements**	**Number (%)**	**Number (%)**
IRBBB	3 (9%)	4 (12%)
Left ventricular hypertrophy	1 (3%)	1 (3%)
Right ventricular strain	4 (12%)	4 (12%)
Any ECG change	24 (73%)	22 (67%)

*DMD* Duchenne muscular dystrophy, *ECG* electrocardiogram, *IRBBB* incomplete right bundle branch block, *LBBB* left bundle branch block, *n* number, *RBBB* right bundle branch block

### Cardiac biomarkers

No clear progression in cardiac biomarker abnormalities was observed over the 7-year follow-up period.

Two women had abnormal Pro-BNP levels at baseline, compared to five at follow-up. The five women with abnormal Pro-BNP at follow-up were not the same women who had myocardial fibrosis or reduced LVEF.

There was no significant difference in CK-MB levels between baseline and follow-up (*p* = 0.906). CK-MB was abnormal in 16 women at both timepoints: 13 were the same individuals, while 3 normalized, and 3 became abnormal.

The CK-MB/CK ratio was calculated only for CK-MB values > 4.0 μg/L. None of the women exceeded the 3% threshold at baseline, while two did so at follow-up. Again, these women did not overlap with those who had fibrosis or reduced LVEF.

Troponin T was abnormal in four women at baseline and in seven women at follow-up.

### Muscle fat fraction and muscle strength

No significant correlation was observed between LVEF and muscle FF (R^2^ = 0.0006,* p* = 0.906). After excluding seven women who had received cardiac medication, the correlation remained insignificant (R^2^ = 0.0022, *p* = 0.847).

We also examined the longitudinal linear correlation between muscle ΔFF and ΔLVEF, which was also nonsignificant (R^2^ = 0.0019, *p* = 0.832). Similarly, changes in VPC/h and SVPC/h did not correlate with muscle ΔFF.

The observed muscle ΔFF between baseline and follow-up ranged from 2 to 31% [26].

To explore this further, we divided participants into two groups based on follow-up muscle strength: those with muscle weakness (n = 12) and those with normal muscle strength (n = 15). Among the 12 women with muscle weakness, only one had an abnormal follow-up LVEF. In contrast, five of the 15 women with normal muscle strength had abnormal LVEF. These findings suggest that there was no significant correlation between muscle strength and LVEF.

## Discussion

This prospective 7-year longitudinal study investigate the cardiac natural history of women with pathogenic *DMD* gene variants. We observed that overall cardiac function, structure, and arrhythmic burden remained relatively stable on a group level, however, subtle yet clinically important changes were observed in a subset, emphasizing the importance of nuanced cardiac surveillance.

### Cardiac magnetic resonance imaging: slow progression

At group level, LVEF remained stable at follow-up. This stability persisted even after excluding those who initiated cardiac medication, suggesting that overt systolic dysfunction may develop more slowly or less frequently in women carrying *DMD* variants compared to affected men, in whom deterioration typically begins earlier and progresses more rapidly [27]^.^ [28].

Six women had LVEF < 56% at baseline; during follow-up, one had a clinically meaningful decline, even received treatment, and two remained stable.

The remaining women remained stable with no clinically meaningful change in LVEF.

These support targeted interval surveillance, with shorter intervals for women with reduced LVEF and extended intervals for those with preserved LVEF.

Several women initiated cardiac medication between baseline and follow-up, which may have contributed to stabilization or improvement of their LVEF.

Myocardial fibrosis remained unchanged in prevalence, with no new cases identified. Fibrosis was consistently localized to the inferolateral or lateral–inferolateral left ventricular wall. However, a few women with pre-existing fibrosis at baseline, LGE appeared visually more extensive at follow-up. As image quality precluded formal quantification, this finding should be interpreted cautiously. Nevertheless, it suggests that fibrosis may become more visually apparent over time even without segment-level extension or an initial parallel decline in LVEF.

### Holter monitoring and electrocardiography: low burden of arrhythmias

Most women did not develop major arrhythmias on Holter monitoring, although there was a measurable increase in ventricular ectopy over time.

Notably, high-burden arrhythmias did not consistently overlap with reduced LVEF or fibrosis, indicating that structural and arrhythmic abnormalities can occur in different individuals.

The median VPC/h frequency in our cohort increased from 0.23 /h (IQR 0.04–1.25) at baseline to 6.38/h (IQR 0.85–15.10) after 7 years. In an older community-based cohort, median PVC frequency increased from 0.5/h (IQR 0.1–4.7) to 1.2/h (IQR 0.1–13.8) over 5 years [29]; thus, the increase observed in our cohort was numerically greater, but direct comparison should be interpreted cautiously given differences in cohort characteristics and follow-up duration.

Importantly, the absolute VPC burden at follow-up remained modest, and high-burden ventricular arrhythmias remained uncommon; therefore, the observed increase is unlikely to be clinically meaningful in most participants.

The change in SVPC/h was smaller and not statistically significant.

In our cohort, the median SVPC/h frequency increased from 0.37/h (IQR 0.13–1.06) to 1.77/h (IQR 0.63–4.98) over 7 years, which is only slightly higher than older healthy adults of similar age [30].

Although our data reflect a slightly higher and faster rate of change than in healthy individuals, the difference does not appear to be clinically significant.

ECG parameters remained largely stable over time.

DMD-specific abnormalities were common but did not progress, and no new conduction blocks or major disturbances developed. These findings suggest that while ECG is useful for baseline assessment, its limited sensitivity to dynamic electrical changes may restrict its utility as a stand-alone monitoring tool.

Together, these findings underscore the importance of rhythm surveillance in these women, which may be most valuable in those with rising ectopy or other arrhythmic abnormalities.

### Cardiac biomarkers: no consistent link to fibrosis or LVEF decline.

Across follow-up, Pro-BNP, CK-MB, and Troponin T showed only slight fluctuations, without any consistent association with fibrosis, reduced LVEF, or change in LVEF.

These findings suggest that commonly used cardiac biomarkers have limited value for tracking subclinical disease progression in women with *DMD* variant, functioning more as adjuncts rather than as primary surveillance tools.

Although still useful for detecting acute myocardial stress, they appear less informative for long-term follow-up in this population.

### Muscle MRI and muscle strength: dissociation from cardiac function

A key observation was the lack of correlation between skeletal muscle involvement, as assessed by both muscle strength and muscle function, and cardiac dysfunction.

Notably, more women with normal muscle strength had abnormal LVEF compared to those with overt muscle weakness, highlighting a paradoxical dissociation between skeletal and cardiac manifestations. One possible explanation for this apparent dissociation is tissue-specific variability in X-chromosome inactivation, which may result in different degrees of dystrophin deficiency in cardiac and skeletal muscle [31]. However, as X-chromosome inactivation was not assessed in the present study, this interpretation remains speculative.

This finding suggests that cardiac involvement may progress silently, even in women without neuromuscular symptoms [32].

Consistent with the previous studies, this dissociation supports the view that skeletal and cardiac involvement can follow distinct and independent trajectories in women carrying *DMD* gene variants⁶⁻⁷. Although muscle weakness often serves as the initial clinical indicator of disease, it cannot be relied upon to predict cardiac health.

Therefore, these findings reinforce a crucial clinical principle: cardiac surveillance is essential for all female carriers, regardless of neuromuscular phenotype. A similar dissociation has been observed in male dystrophinopathies [33].

### Limitations

The 36% of nonparticipation may have introduced a potential risk of selection bias. However, we compared baseline characteristics of participants and nonparticipants, age, BMI, genotype, and baseline cardiac status (reduced LVEF, fibrosis, any Holter/ECG abnormality) were similar between groups. Nonetheless, residual selection bias cannot be excluded.

Secondly, despite comprehensive multimodal assessments, practical constraints led to incomplete datasets for some participants, particularly for CMR and Holter monitoring.

Finally, changes in equipment and assay methods—such as CMR scanner upgrades—between time points may limit comparability of measurements.

## Conclusion

Over seven years, women carrying pathogenic *DMD* gene variants have stable left ventricular function at group level; a minority showed within-person decline. Pre-existing myocardial fibrosis progressed in a few. High-burden or sustained arrhythmias were uncommon.

ECG intervals were largely unchanged.

These findings support targeted surveillance rather than blanket frequent monitoring: follow-up should be prioritized in those with symptoms, rising ectopy, reduced LVEF, or existing fibrosis. Extended intervals of cardiac follow-up should be considered in asymptomatic women with normal baseline results.

## Data Availability

The datasets generated and/or analyzed during the current study are not publicly available due to data protection regulations and patient privacy but are available from the corresponding author on reasonable request.

## Abbreviations

AVB: Atrioventricular conduction block
BMD: Becker muscular dystrophy
CI: Confidence interval
CK: Creatine kinase
CMR: Cardiac magnetic resonance
DMD: Duchenne muscular dystrophy
*DMD* gene: Dystrophin gene
ECG: Electrocardiography
ECV: Extracellular volume
eGFR: Estimated glomerular filtration rate
FF: Fat fraction
ILBBB: Incomplete left bundle branch block
IQR: Interquartile range
IRBBB: Incomplete right bundle branch block
LA: Left atrial
LBBB: Left bundle branch block
LGE: Late gadolinium enhancement
LVEDV: Left ventricular end-diastolic volume
LVEF: Left ventricular ejection fraction
MBH: Maria Buhl Hjortrup
MOLLI: Modified Look-Locker inversion recovery
MRC: Medical Research Council
MRI: Magnetic resonance imaging
N: No
NA: Not available
NGV: Niels Grove Vejlstrup
NSP: Nanna Scharff Poulsen
NSVT: Nonsustained ventricular tachycardia
Pro-BNP: Pro-brain natriuretic peptide
RBBB: Right bundle branch block
RV: Right ventricle
RVEDV: Right ventricular end-diastolic volume
RVEF: Right ventricular ejection fraction
SD: Standard deviation
SVPC/h: Supraventricular premature contractions per hour
SVT: Supraventricular tachyarrhythmias
VPC/h: Ventricular premature contractions per hour
Y: Yes
y: Year
ZL: Zhe
ΔEF: Follow-up FF − baseline FF
ΔLVEF: Follow-up LVEF − baseline LVEF
